# Monofloral Triadica Cochinchinensis Honey Polyphenols Improve Alcohol-Induced Liver Disease by Regulating the Gut Microbiota of Mice

**DOI:** 10.3389/fimmu.2021.673903

**Published:** 2021-05-21

**Authors:** Liping Luo, Jinping Zhang, Mingyan Liu, Shengrong Qiu, Shengxiang Yi, Wenjie Yu, Tao Liu, Xueyong Huang, Fangjian Ning

**Affiliations:** ^1^ School of Life Sciences, Nanchang University, Nanchang, China; ^2^ State Key Laboratory of Food Science and Technology, Nanchang University, Nanchang, China

**Keywords:** *Triadica Cochinchinensis*, honey, polyphenols, alcohol-induced liver disease, gut microbiota

## Abstract

Honey produced from medicinal plants holds great promise for human health. Increasing evidence suggests that the gut microbiota plays an important role in liver pathology after alcohol intake. The aim of this study was to identify the polyphenol composition of *triadica cochinchinensis* honey (TCH), and to study the potential effect of honey polyphenols on the regulation of gut microbes in mice with alcohol-induced liver injury and the improvement of alcohol-induced liver disease. For these purposes, a total of 190 compounds were identified and 27 of them were quantified by ultraperformance liquid chromatography coupled with quadrupole/time-of-flight mass spectrometry (UPLC-Q/TOF-MS) and we successfully established a mouse model of alcohol-induced liver injury. The results show that TCH polyphenols can significantly restore the levels of ALT and AST, and TCH intervention can significantly improve the pathological changes of liver tissue in alcohol-exposed mice. Additionally, a significant decrease was observed in Firmicutes/Bacteroidetes after TCH treatment. Moreover, KEGG pathways of ATP-binding cassette (ABC) transporters, two-component system and biosynthesis of amino acids enriched the most differentially expressed genes after TCH intervention for 8 weeks. Our results may have important implications for the use of TCH as a functional food component with potential therapeutic utility against alcohol-induced liver disease.

## Introduction

Alcoholic liver disease (ALD) is a type of alcohol-induced chronic progressive liver disease (1). Alcohol-related liver cirrhosis accounts for 0.9% of global deaths and 47.9% of all deaths from cirrhosis-related diseases, which seriously threatens human health (2). The progression of ALD includes multiple stages, from steatohepatitis, alcoholic hepatitis, liver fibrosis, cirrhosis, and even liver cancer (3). At this stage, there is still a lack of effective drug treatments for ALD. The possible mechanisms of ALD are mainly alcohol metabolism-related oxidative stress damage, abnormal methionine metabolism, intestinal flora imbalance and bacterial translocation, inflammatory mediator damage, and nutritional imbalance, etc. Among them, the intestinal flora imbalance is considered to be an important factor in the occurrence and development of ALD, which has attracted wide attention from scholars at home and abroad (4, 5).

The gut microbes are a large number of microorganisms that colonize the human digestive tract with complex types. The total weight of the intestinal flora of healthy adults is 1 to 2 kg, the number is at least 1 × 10^14^, which is 10 times the number of human cells, and the number of genes is 150 times the number of human genes (6, 7). Studies have found that the gut microbes are involved in multiple stages of host material metabolism, immune defense, growth and development, and the imbalance of intestinal flora is related to many diseases, such as colon cancer, liver cirrhosis, diabetes, hypertension, autoimmunity Diseases, etc. (8, 9). Gut microbes have a significant imbalance in ALD patients and play an important role in the occurrence of ALD. The structure and function of the intestinal flora have changed in patients with moderate drinking, alcohol abuse and alcoholic liver cirrhosis (10, 11).

Plant polyphenols are a class of polyhydroxy compounds, which are secondary metabolites with a polyphenol structures widely found in tea, grapes, apples, wine, and a variety of fruits and vegetables (12). Because of its antioxidant, anti-tumor, liver protection, and anti-obesity functions, plant polyphenols have a wide range of applications in the food industry (13, 14). With the development and deepening of related theoretical research, researchers have found that plant polyphenols have a greater effect on maintaining the homeostasis of the intestinal microenvironment. So far, many test results have proved that polyphenols can inhibit the growth and reproduction of harmful bacteria in the intestine, promote the growth of beneficial bacteria such as Lactobacillus and Bifidobacterium, and optimize the structure of the intestinal flora. Therefore, researchers have regarded polyphenols as the third major regulator of intestinal health besides prebiotics and probiotics (15, 16).

*Triadica cochinchinensis Loureiro* is a tree or shrub of the genus Tricadica in the Euphorbiaceae family. It is widely distributed in southern China, South Asia, and Southeast Asia (17). Early studies have shown that *triadica cochinchinensis* leaves can be used to treat skin diseases, such as eczema, dermatitis, scabies, and shingles, and the roots can be used to treat constipation dysuria, edema, and wounds (18). Recent studies have found that *triadica cochinchinensis* tree contains Coumarinolignoids (coumarin), which has anti-inflammatory, antioxidant, and hepatoprotective effects. It also contains taraxeranes, which have cytotoxic and allelopathic effects (19). Qinpiting and ent-kaurane-3-oxo-16α, 17-diol in the stems and leaves of *triadica cochinchinensis* tree have a significant inhibitory effect on the release of NO from BV-2 microglia activated by LPS. So far, the composition and efficacy of nectar secreted by *triadica cochinchinensis* tree have not been studied (20).

Honey is a natural product produced by honey bees from nectar or secretions originally gathered from flowering plants. Although the predominant constituents of honey are fructose and glucose, the minor components, such as amino acids, organic acids, vitamins, minerals, enzymes, and polyphenols, endow honey with distinct colors, flavors, and therapeutic effects (21). Previous studies have reported on the effectiveness and medical application of honey against various diseases, such as antimicrobial, anti-cardiovascular, anticancer, and antidiabetic effects (22). In particular, phenolic compounds constitute an important class of biologically active compounds that act as antioxidants and scavenging free radicals. Cao reports that A. cerana honey, gathered from Apis cerana Fabricius (A. cerana), had high total phenolic contents (345.1-502.1 mg GA kg-1), ascorbic acid contents (153.8-368.4 mg kg-1), could prevent acute alcohol-induced liver damage likely because of its antioxidant properties and ability to prevent oxidative stress (23). Wang reported monofloral honey from a medical plant, Prunella Vulgaris, protected against dextran sulfate sodium-induced ulcerative colitis *via* modulating gut microbial populations in rats (24). Therefore, identifying the primary polyphenolic compounds in honey from the medicinal plant *triadica cochinchinensis* would help maximize its health benefits and commercial value. However, to our knowledge, there is no information reported on *riadica cochinchinensis* honey (TCH)

The purpose of this study was to determine the polyphenol components of TCH that may contribute to most biological activity. Furthermore, we explored the potential protective effects of TCH against alcohol-induced liver injury in mice. The results of this study provide fundamental data for the study of TCH. It may also help to improve the recognition of the health benefits and commercial value of TCH.

## Materials and Methods

### Materials and Reagents

Alanine aminotransferase (ALT) determination kit and Aspartate aminotransferase (AST) determination kit were purchased from Changchun Huili. Ethanol (HPLC grade), silymarin, glucose, fructose were purchased from Sigma-Aldrich (St. Louis, MO, USA). LC/MS-grade acetonitrile, methanol, formic acid, and water were purchased from Merck (Darmstadt, Germany). *Riadica cochinchinensis* honey (TCH) was taken from Anfu County, Ji’an City, Jiangxi Province.

### UPLC-Q/TOF-MS Analysis of TCH

Analysis of the samples was performed on a Waters ACQUITY UPLC System (Waters, Milford, MA, USA) equipped with an HSS T3 column (2.1×100 mm, 1.8 µm; Waters, Milford, MA, USA), which was controlled by Masslynx 4.1 software. The mobile phases were water with 0.02% formic acid (A) and acetonitrile with 0.02% formic acid (B) (V/V) with the following linear gradient elution: 0-2 min, 5-10% B; 2-5 min, 10-40% B; 5-10 min, 40-70% B; 10-12 min, 70-80% B; 12-14 min, 80-80% B; 14-15 min, 80-100% B; 15-17 min, 100-100% B; 17-17.5 min, 100-5% B; 17.5-21 min, 5-5% B. The flow rate was maintained at 0.4 mL/min, and the column was operated at 40°C. The injection volume was set to 5 μL.

The UPLC system was connected to Waters Xevo G2-XS Q/TOF (Waters, Milford, MA, USA) equipped with a Z-Spray ESI source. Both positive and negative ionization modes were carried out with a mass range of 50-1200 Da with a scan time of 0.2 s. MS conditions were set as follows: 3.0 kV for positive and 2.5 kV for negative, capillary voltage; 40 V, sampling cone voltage; 20°C, desolvation temperature; 120°C, source temperature; 800 L/h, desolvation gas flow rate; 50 L/h, cone gas flow rate. MS^e^ data were obtained in centroid mode with a mass range 50-1200 Da both in low-energy (function 1) and high-energy (function 2) scan functions. For the low-energy scan function, collision energy was 6 V, and scan time was 0.2 s. For the high-energy scan function, a collision energy ramp of 10-45 V was used with a scan time of 0.2 s. Leucine-enkephalin (0.1 µg/mL) was used as lock-mass solution. This solution was introduced by LockSpray at 10 µL/min and used to generate the reference ions at *m*/*z* 556.2766 (positive mode) and *m*/*z* 554.2620 (negative mode). The quantification and calibration curves of the main compounds were generated and calculated by plotting the calibration compound standards against corresponding peak areas

### Animals and Experimental Design

Fifty-Six male C57BL/6J mice (6 weeks old, weighing 18 g-22 g.) were obtained from Hunan Slack Jingda Experimental Animal Co., Ltd. (Nanchang, China, permission number: SYXK(G)2015-0002) and housed in cages under 25 ± 2°C and a 12 h light/12 h dark cycle with 50 ± 10% relative humidity throughout the study. Feed and water were provided without restriction for 1 week before the experiments began. 56 mice were randomly divided into 7 groups, each with 8 (1): Pair fed control group (PF) (2) alcohol-fed model group (AF) (3) positive control group (silymarin, 0.05 g/Kg body weight, PC) (4) Low-dose TCH group (5 g/Kg body weight, LH) (5) Middle-dose TCH group (10 g/Kg body weight, MH) and (6) High-dose TCH group (20 g/Kg body weight, HH) (7) Fructose syrup group (20 g/Kg body weight, FG). The sample group was given different doses of TCH every day, the positive control group was given silymarin, the blank group and the alcohol model group were given equal volumes of distilled water, and the fructose syrup group was given a prepared fructose syrup (fructose: glucose=48:32), experiment for 12 consecutive weeks (25). In the 13th week, all groups except the blank group were given 5% ethanol (v/v) for 10 consecutive days, with 30 mL in each group. On the 11th day, 31.5% ethanol (v/v) was given by gavage. The blank control group was given the same amount of normal saline, and the gavage amount was 5 g/Kg (26).

After the last treatment, the mice were fasted with water for 12 hours and weighed and recorded their body weight. The mice were anesthetized with 100 μL of 10% chloral hydrate, and 1 mL of blood was taken from the orbit, and EDTA-2K was used for anticoagulation. The whole liver was dissected and taken out, rinsed repeatedly in normal saline at 4°C, the filter paper absorbed water, and liver tissue of a certain size (2 cm × 0.5 cm × 0.4 cm) was cut out and fixed with 10% formalin solution.

### Serum Physiological Indicators and Liver Histopathological Examination

After the blood is allowed to stand, centrifuge at low speed at 4°C to obtain the upper serum. Use ALT and AST kits to detect the content of ALT and AST in serum. The livers were fixed in a 4% paraformaldehyde solution for 48 h, then dehydrated in a graded series of ethanol solutions, embedded in paraffin, and sectioned into 4 μm thick slices. These slices were stained with hematoxylin and eosin (H&E). Histological images were taken by the microscopy imaging system (Leica DM1000, Nussloch, Germany)

### Gut Microbiota Analysis

The contents of the mouse colon were taken in a sterile cryopreservation tube, quickly frozen in liquid nitrogen, and then transferred to a -80°C refrigerator for low-temperature storage.

Microbial community genomic DNA was extracted from samples using the E.Z.N.A.^®^ soil DNA Kit (Omega Bio-tek, Norcross, GA, U.S.) according to the manufacturer’s instructions. The DNA extract was checked on 1% agarose gel, and DNA concentration and purity were determined with NanoDrop 2000 UV-vis spectrophotometer (Thermo Scientific, Wilmington, USA). The hypervariable region V3-V4 of the bacterial 16S rRNA gene were amplified with primer pairs 338F (5’-ACTCCTACGGGAGGCAGCAG-3’) and 806R (5’-GGACTACHVGGGTWTCTAAT-3’) by an ABI GeneAmp^®^ 9700 PCR thermocycler (ABI, CA, USA). The PCR amplification of 16S rRNA gene was performed as follows: initial denaturation at 95°C for 3 min, followed by 27 cycles of denaturing at 95°C for 30 s, annealing at 55°C for 30 s and extension at 72°C for 45 s, and single extension at 72°C for 10 min, and end at 4°C. The PCR mixtures contain 5 × TransStart FastPfu buffer 4 μL, 2.5 mM dNTPs 2 μL, forward primer (5 μM) 0.8 μL, reverse primer (5 μM) 0.8 μL, TransStart FastPfu DNA Polymerase 0.4 μL, template DNA 10 ng, and finally ddH_2_O up to 20 μL. PCR reactions were performed in triplicate. The PCR product was extracted from 2% agarose gel and purified using the AxyPrep DNA Gel Extraction Kit (Axygen Biosciences, Union City, CA, USA) according to manufacturer’s instructions and quantified using Quantus™ Fluorometer (Promega, USA).

Purified amplicons were pooled in equimolar and paired-end sequenced (2 ×300) on an Illumina MiSeq platform (Illumina, San Diego, USA) according to the standard protocols by Majorbio Bio-Pharm Technology Co. Ltd. (Shanghai, China). The raw reads were deposited into the NCBI Sequence Read Archive (SRA) database.

### Processing of Sequencing Data

The raw 16S rRNA gene sequencing reads were demultiplexed, quality-filtered by Trimmomatic, and merged by FLASH with the following criteria: (i) the 300 bp reads were truncated at any site receiving an average quality score of <20 over a 50 bp sliding window, and the truncated reads shorter than 50 bp were discarded, reads containing ambiguous characters were also discarded; (ii) only overlapping sequences longer than 10 bp were assembled according to their overlapped sequence. The maximum mismatch ratio of the overlap region is 0.2. Reads that could not be assembled were discarded; (iii) Samples were distinguished according to the barcode and primers, and the sequence direction was adjusted, exact barcode matching, 2 nucleotide mismatch in primer matching.

Operational taxonomic units (OTUs) with 97% similarity cutoff were clustered using UPARSE (version 7.1, http://drive5.com/uparse/), and chimeric sequences were identified and removed. The taxonomy of each OTU representative sequence was analyzed by RDP Classifier (http://rdp.cme.msu.edu/) against the 16S rRNA database (e.g. Silva SSU128) using a confidence threshold of 0.7.

## Results and Discussion

### Compound Profile of TCH

**Figure 1:**
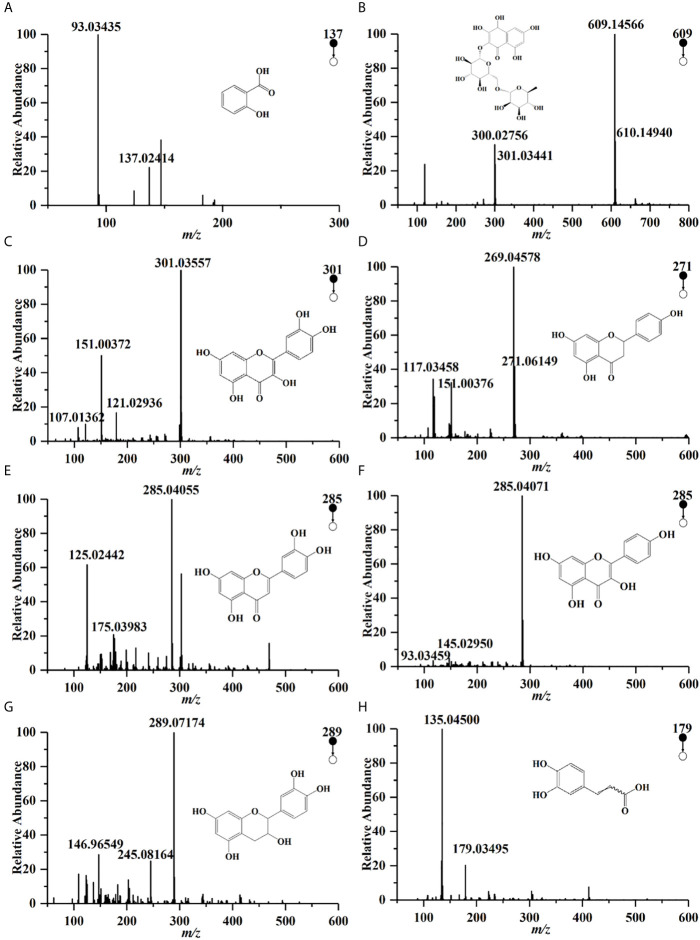
UPLC-Q/TOF-MS spectra of some polyphenols in HCT in negative ion pattern: **(A)** salicylic acid; **(B)** rutin; **(C)** quercetin; **(D)** narigenin; **(E)** luteolin; **(F)** kaemperol; **(G)** epicatedin; **(H) **caffeic acid.

Compounds profiles of TCH were obtained by UPLC-Q/TOF-MS analysis (**Supplementary Figure 1**). A total of 190 compounds were identified in negative ionization mode (Mass error ≤ 5mDa). Among the 190 components, a total of 27 compounds were identified and quantified by comparing retention times and MS spectra with available standards (**Table 1**). While others were confirmed by exact molecular mass and their MS fragments with available standards, reported literature, or the public databases of ChemSpider (http://www.chemspider.com/) and MassBank (http://www.massbank.jp/). For example, two organic acids were detected at the negative ionization mode. The dominating product ions of these compounds corresponded to the loss of water molecules (m/z 18.0) and carbon dioxide (m/z 44.0). According to literature and available standards, compounds (**Figures 1A, H**) were proposed as salicylic acid, caffeic acid, respectively. In accordance with the reports, compounds **Figures 1B–F** were proposed as rutin, quercetin, narigenin, luteolin and kaemperol, respectively. Rutin was suggested for the precursor ion at m/z 609.14566, and produced a fragment ion [M−H]− at 301.03441 (quercetin). The total polyphenols content of TCH was 23.4 mg GAE/100 g.

**Table 1:** Compounds detected in Citrus nectars and honeys in negative ion mode.

Pear No	Compound	Formula	CAS	RT(min)	Theoretical (m/z negative)
1*	Gallic acid	C_7_H_6_O_5_	149-91-7	1.39	169.0129
2*	3,4-Dihydroxybenzoic acid	C_7_H_6_O_4_	99-50-3	2.29	153.0188
3*	Salicylic acid	C_7_H_6_O_3_	69-72-7	3.16	137.0245
4*	Chlorogenic acid	C_16_H_18_O_9_	327-97-9	3.3	353.0873
5*	Epicatechin	C_15_H_14_O_6_	35323-91-2	3.36	290.0752
6*	Caffeic acid	C_9_H_8_O_4_	331-39-5	3.66	179.0344
7*	Rutin	C_27_H_30_O_16_	153-18-4	4.23	609.1456
8*	Ellagic acid	C_14_H_6_O_8_	476-66-4	4.33	300.9985
9*	Genistein 7-O-glucoside	C_21_H_20_O_10_	529-59-9	4.46	432.1019
10	Isorhamnetin-3-O-glucoside	C_22_H_22_O_12_	5041-82-7	4.58	477.1034
11*	p-Hydroxybenzoic acid	C_7_H_6_O_3_	99-96-7	4.85	137.0239
12*	Fisetin	C_15_H_10_O_6_	528-48-3	4.98	285.0399
13	Dihydrokaempferol	C_15_H_12_O_6_	480-20-6	5.03	287.0556
14*	Methyl syringate	C_10_H_12_O_5_	884-35-5	5.24	212.0647
15*	3,4-Dimethoxycinnamic acid	C_11_H_12_O_4_	2316-26-9	5.3	207.0657
16*	Abscisic acid	C_15_H_20_O_4_	14375-45-2	5.45	263.1291
17*	Luteolin	C_15_H_10_O_6_	491-70-3	5.49	285.0399
18*	Quercetin	C_15_H_10_O_7_	117-39-5	5.52	301.0348
19	Pinobanksin-5-methyl ether	C_16_H_14_O_5_	119309-36-3	5.74	285.0758
20*	Naringenin	C_15_H_12_O_5_	480-41-1	5.98	271.0606
21*	Apigenin	C_15_H_10_O_5_	520-36-5	5.99	269.0450
22*	Kaempferol	C_15_H_10_O_6_	520-18-3	6.08	285.0399
23	Luteolin 5-methyl ether	C_16_H_12_O_6_	58115-29-0	6.12	299.0561
24	3-O-Acetylpinobanksin	C_17_H_14_O_6_	52117-69-8	6.29	314.0714
25*	Phenethyl caffeate	C_17_H_16_O_4_	104594-70-9	7.71	283.0973
26*	Biochanin A	C_16_H_12_O_5_	491-80-5	7.77	283.0606
27	Galangin-5-methyl ether	C_16_H_12_O_5_	104594-69-6	7.95	283.0613

*Confirmed using standards.

### Influence of TCH on Serum Physiological Indicators and Liver Histopathological of Alcoholic-Induced Liver Damage Obesity Mice Model

**Figure 2:**
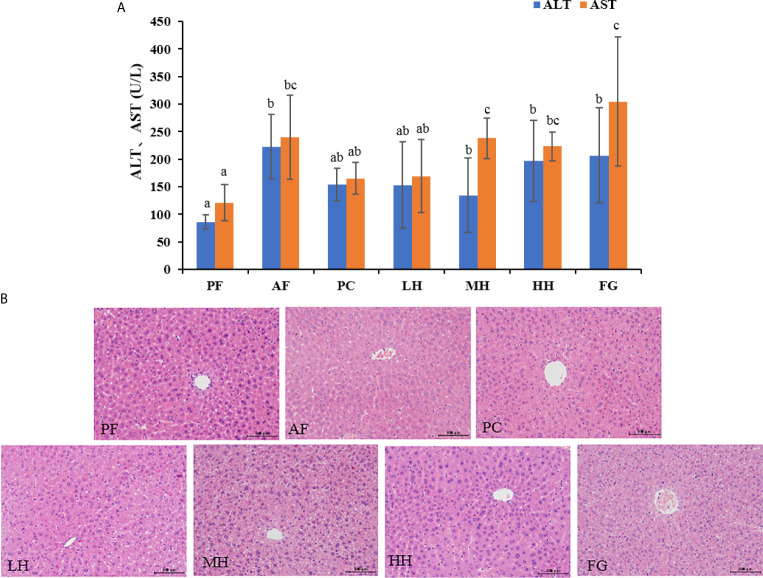
**(A)** Effects of TCH on serum ALT and AST. Different lowercase letters indicate significant difference (P < 0.05). **(B)** Photomicrographs of liver sections stained with hematoxylin-eosin (HE × 400).

Serum ALT and AST levels are important indicators of liver injury. **Figure 2A** shows the ALT and AST in the serum of mice in each group. The ALT and AST values of the PF group were the lowest, the values of the AF group were significantly higher than those of the PF group, and the ALT and AST values of the PC group were lower than those of the AF group, and there was no significant difference. The values of ALT and AST of each group of honey are lower than those of AF group, and the value of LH in honey group is the lowest.

The pathological changes of mouse liver tissue were observed by HE staining method. As shown in the **Figure 2B**, the liver cells of the PF group are complete, the nucleus is clear, and the liver cords are arranged neatly; while the liver cords of the AF group are disorderly arranged, the outline of liver cells is blurred, and there are obvious hepatocyte edema and steatosis, accompanied Inflammatory cell infiltration; honey intervention can significantly improve the pathological changes of liver tissue in alcohol-exposed mice, showing that the mouse liver cell edema and steatosis are alleviated, the liver cords are arranged more neatly, and the inflammatory cell infiltration is reduced. The PC group had a more obvious improvement than AF, and the FG group had more severe liver damage than the AF group. **Supplementary Table 1** shown the histopathological assessment grading, the basic pathological changes of alcoholic liver disease are classified into three major items, namely steatosis, inflammation and fibrosis, and each item is divided into grades 1-4 according to the degree. The liver tissue of the PF group was normal and there was no disease; the liver cell steatosis of the AF group was more than 75%, and there was a certain degree of inflammatory cell infiltration; the PC group had mild liver steatosis and inflammation; the LH, MH and HH groups had steatosis and inflammation In terms of alcoholic liver injury, it has a certain alleviating effect; the FG group aggravated the alcoholic liver injury, the degree of liver steatosis was more serious than that of the AF group, and inflammatory cell infiltration, small-scale liver fibrosis, liver damage was the most serious.

### Influence of TCH on Gut Microbiota Diversity of Mice Model

**Figure 3:**
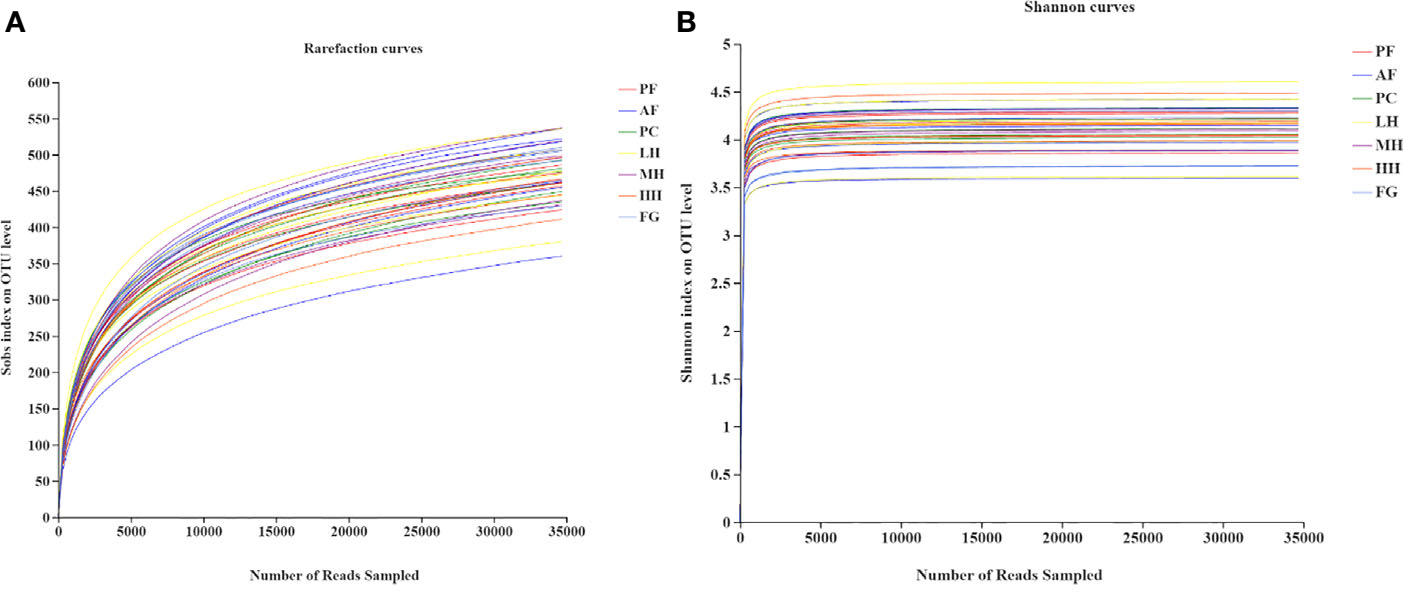
Rarefaction analysis **(A)** and Shannon index **(B)** of gut microbiota from seven groups of mice.

Analysis of the dilution curve (**Figure 3**) of the gut microbiota sequencing sample shows that this sequencing has basically covered all species in the sample. Dilution curve is to randomly select a certain number of individuals from a sample, count the number of species represented by these individuals, and construct a curve based on the number of individuals and species. It can be used to compare the abundance of species in samples with different amounts of sequencing data, and it can also be used to show whether the amount of sequencing data of a sample is reasonable. The method of a random sampling of sequences is used to construct a dilution curve based on the number of sequences drawn and the number of OTUs they can represent. When the curve tends to be flat, it indicates that the amount of sequencing data is reasonable, and more data will only generate a small amount of new data. On the contrary, it indicates that more new OTUs may be generated by continuing to sequence. Therefore, as known from the dilution curve of this experiment, the depth of this sequencing has basically covered all species in the sample. Moreover, it can be seen from the Shannon index curve of this experiment that the plateau period has been reached, indicating that this experiment has a good degree of sample species richness under the existing sequencing depth, which can meet the requirements for further analysis.

Alpha diversity can reflect the abundance and uniformity of microbial communities, including Chao index, Ace index, Shannon index, and Simpson index. Among them, the Chao index and Ace index reflect the abundance of species in the community. The larger the Chao or Ace index, the higher the community abundance. The Shannon index and Simpson index reflect the diversity of the community. The larger the index, the higher the diversity of the community; the larger the Simpson index, the lower the diversity of the community. As shown in **Supplementary Figure S2**, there is no significant difference in the species abundance and diversity of the intestinal flora between the groups. The Chao index and Ace index show that alcohol increases species abundance, and honey may reduce the increase in abundance caused by alcohol, especially low-concentration honey. Fructose syrup will increase species abundance compared to honey. Shannon index and Simpson index show that alcohol increases the species diversity of the intestinal flora, high concentrations of honey can also increase species diversity, and fructose syrup reduces species diversity.

**Figure 4:**
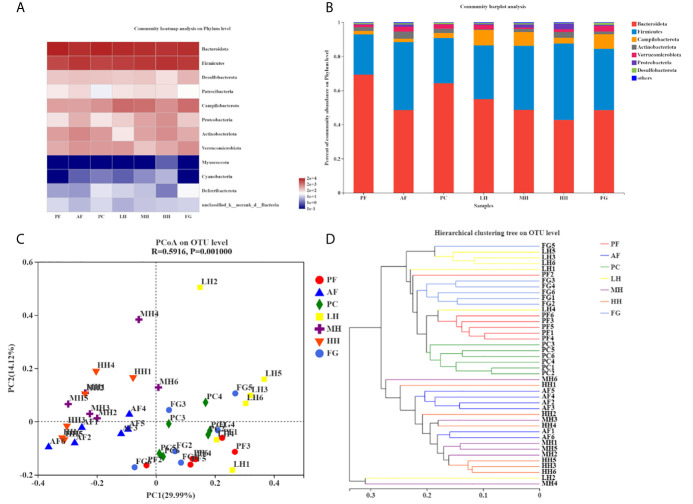
TCH alters gut microbiota structure in mice. Structures of gut microbiota of mice were analyzed using 16S rRNA gene sequencing and bioinformatics analysis. The relative Community heatmap analysis on phylum level **(A)**. The composition and relative abundances of the gut microbiota at the phylum level **(B)**. Principal coordinates analysis (PCoA) plots based on unweighted UniFrac **(C)**. Hierarchical clustering tree on OUT level **(D)**.

**Table 2** corresponds to **Figures 4A, B**, showing the proportion of dominant species in the gut microbiota of each group of mice. **Figures 4A, B** show the species abundance of each group at the phylum level. The species with higher abundance in each group mainly include *Bacteroidota, Firmicutes, Campilobacterota, Actinobacterota, Verrucomicrobiota, Proteobacteria, Desulfobacterota.* The Bacteroidota and Campilobacterota of AF are lower than the PF group, and Firmicutes, *Actinobacteriota, Verrucomicrobiota, Proteobacteria* and *Desulfobacterota* are higher than the PF group. The results of the PC group and the PF group were not much different. Both LH and MH can increase the abundance of Bacteroidota due to alcohol in the AF group, and reduce the abundance of Firmicutes in the AF group due to alcohol. In the HH group, Bacteroidota and Firmicutes, the two gates, may have aggravated the effect of alcohol. There is little difference between the results of FG and AF groups.

**Table 2:** The composition table of dominant species in each group of mouse intestinal flora phylum level.

Phylum	PF	AF	PC	LH	MH	HH	FG
Bacteroidota	69.34%	48.62%	64.31%	54.96%	48.71%	42.75%	48.55%
Firmicutes	23.54%	39.78%	26.45%	31.49%	37.49%	44.85%	35.94%
Campilobacterota	2.07%	1.98%	3.01%	9.10%	8.10%	3.28%	8.60%
Actinobacteriota	2.19%	4.18%	2.50%	0.29%	1.89%	3.47%	1.51%
Verrucomicrobiota	1.49%	2.86%	2.45%	2.59%	0.77%	1.57%	3.31%
Proteobacteria	0.33%	1.30%	0.30%	0.57%	1.85%	3.31%	0.64%
Desulfobacterota	0.70%	0.79%	0.75%	0.63%	0.73%	0.36%	1.12%
Firmicutes/Bacteroidetes ratio (F/B)	33.95%	81.82%	41.13%	57.30%	76.97%	104.91%	74.03%

PCoA analysis, namely principal coordinate analysis, is a method to study the similarity and difference of data. The larger the R, the higher the explanation of the difference between the groups; the P value is less than 0.05, the higher the reliability of this test. It can be seen from the PCoA diagram (**Figure 4C**) that the structure of the gut microbes of each group of mice presents distinct clusters. AF and PF, PC, LH, and FG are far apart and do not overlap, and the community composition is quite different; AF and MH, HH is relatively close, and some overlap, and the community composition is relatively similar; LH is far away from MH and HH, and the community composition is very different; HH is far from FG, and the difference is large; PF is closer to PC, LH, and FG. And some overlap, the community composition is relatively similar.

Sample hierarchical cluster analysis: Use bray-curits algorithm to perform hierarchical cluster analysis on all samples, draw a tree diagram according to the similarity between samples, and reflect the composition of the bacterial community in the sample. In the tree diagram, the smaller the difference between samples, the closer the branches. Each group is distributed in different branches on the clustering tree, and most of the samples in the group are distributed in clusters (**Figure 4D**). The distance between PF and AF is relatively long, and the difference is large; the distance between PF and PC and FG is relatively short, and the difference is small; the distance between AF and LH is long, and the difference is large; the distance between AF and MH, HH is relatively close, and the difference is small; the distance between HH and FG is long, The difference is large, which is mutually confirmed with the PCoA analysis results.

**Figure 5:**
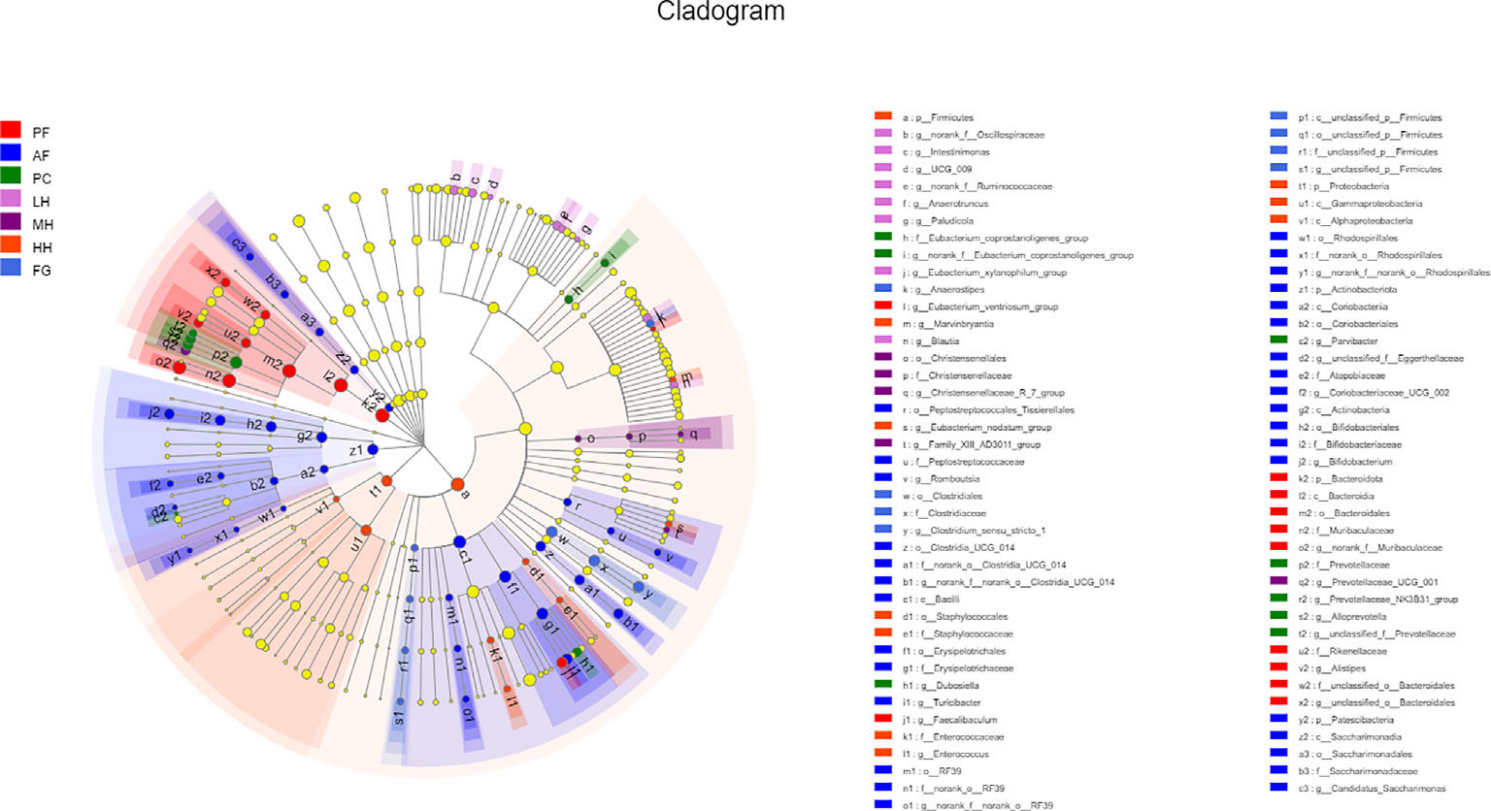
LEfSe analyses of gut microbiota in seven groups of mice. Differentially expressed taxa with the LDA scores > 2.0 and adjusted p < 0.05. The taxonomic cladogram shows the relative abundance of OTUs with circles representing phylogenetic levels from phylum (innermost circle) to species (outermost circle) and the diameter of each circle being proportional to the taxon’s abundance.

In order to further evaluate the effect of honey on the composition of the gut microbiota of alcohol-fed mice, we used LEFse analysis to identify specifically altered bacterial phenotypes (LDA>2) (**Figure 5**, **Supplementary Figure 3**). There are 83 species of specific species, with 31 species being the most in the AF group, followed by the HH group with 14 species. PF, PC, LH, MH and FG are 11, 8, 7, 4 and 8 in order. There are 5 specific species at the phylum level, namely: *Bacteroidota, Actinobacteriota, Patescibacteria, Firmicutes, Proteobacteria*. There are 8 specific species at the class level, namely: *Bacteroidia, Bacilli, Actinobacteria, Saccharimonadia, Coriobacteriia, Gammaproteobacteria, Alphaproteobacteria, unclassified_p:Firmicutes.* At the order level, there are 14 specific species, namely: *Bacteroidales, Erysipelotrichales, Bifidobacteriales, Clostridia_UCG_014, Rhodospirillales, Saccharimonadales, Coriobacteriales, Peptostreptococcales_Tissierellales, RF39, Christensencales, Enterobacterellales, RF39, Christensencales, Enterobacteriales*. There are 19 specific species at the family level, namely: *Muribaculaceae, Rikenellaceae, unclassified_o:Bacteroidales, Erysipelotrichaceae, Bifidobacteriaceae, norank_o:Clostridia_UCG_014, norank_o:Rhodospirillales Peptostreptococcaceae, Saccharimonadaceae, Atopobiaceae, norank_o:RF39, Prevotellaceae, Eubacterium coprostanoligenes_group, Christensenellaceae, Enterobacteriaceae, Enterococcaceae, Staphylococcaceae, Clostridiaceae, unclassified_p:Firmicutes* and there are 19 specific species at the family level.

### Influence of TCH on the Humanized Mice Gut Microbiome

**Figure 6:**
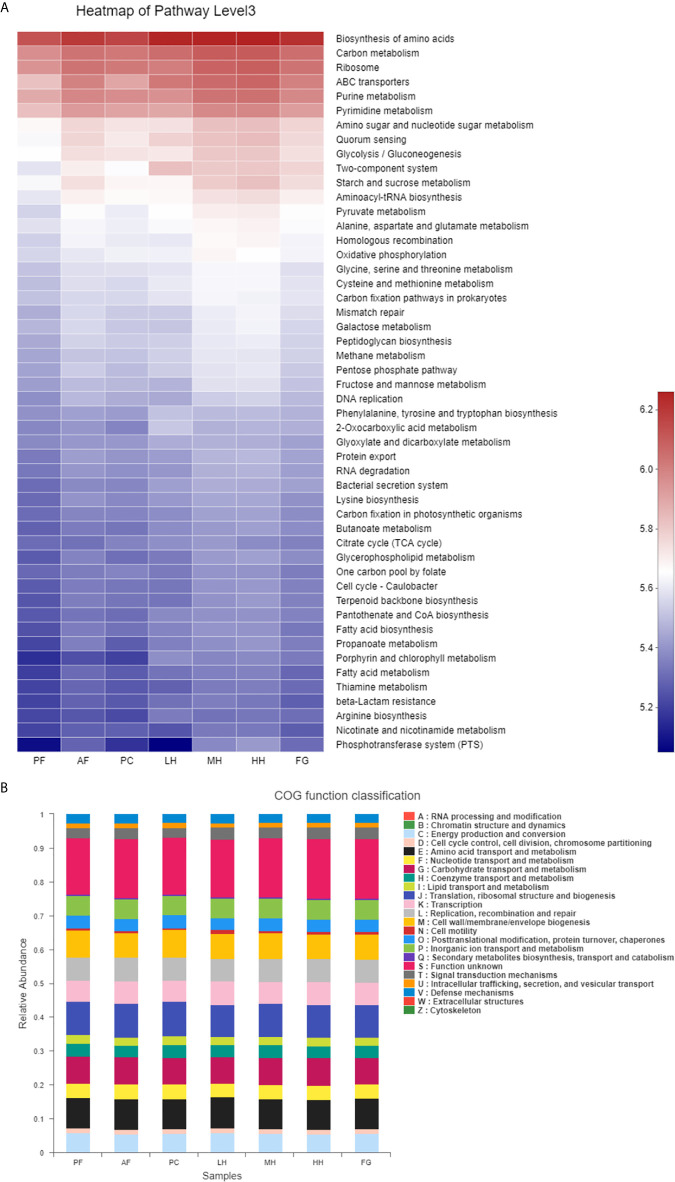
Heatmap of key pathways in KEGG database at level 3 based on PICRUSt predicted functions of the gut microbiota, the colors range from blue to red **(A)**. The relative abundances of the gut microbiota in COG function classification **(B)**.

In order to evaluate the effect of honey on the potential metabolic pathways of the intestinal flora of alcohol-fed mice, we used the PICRUSt analysis method and predicted the metabolic function of the gut microbiota of mice based on the KEGG and COG databases. According to **Figure 6A**, Glycolysis/Gluconeogenesis, Starch and sucrose metabolism, Fruit and mannose metabolism, Galactose metabolism and other metabolism related to sugar metabolism and Ribosome, Purine metabolism, Pyrimidine metabolism, Amino sugar and nucleotide sugar metabolism, Aminoacyl-tRNA biosynthesis, Homologous recombination Metabolism related to protein production, such as Mismatch repair, is increased under the action of alcohol, LH will reduce the metabolic level, and the metabolic level of MH, HH, and FG cannot be reduced, and remains unchanged or increased. According to **Figure 6B**, under the action of alcohol, the metabolism related to protein production, such as Translation, ribosomal structure and biogenesis, RNA processing and modification, Replication, recombination and repair, Chromatin structure and dynamics, Posttranslational modification, protein turnover, chaperones, Nucleotide transport and metabolism, etc., Carbohydrate transport and metabolism sugar metabolism and Lipid transport and metabolism (lipid transport and metabolism) increase, LH will reduce the level of metabolism, the level of metabolism of MH, HH and FG cannot be reduced, the level or increase.

Therefore, the above research results show that alcohol can cause disorders of the intestinal flora of mice, and low concentration of TCH can partially reverse the function of the gut microbiota of AF mice, making it reach a level similar to that of PF mice, So as to improve the metabolic function of alcohol-fed mice.

## Discussion

BAJAJ et al. proposed to reflect the changes of “good bacteria” and “bad bacteria” in the intestinal flora of patients with the liver disease through the cirrhotic flora imbalance index. This ratio is calculated by dividing the number of beneficial intestinal tracts of *Lachnospiraceae, Ruminococcaceae*, and Clostridiales Family XIV Incertae sedis by the number of potentially pathogenic bacteria *Enterobacteriaceae* and *pseudobacteriaceae.* The number of Bacteroidaceae. In experimental animals and clinical studies, long-term alcohol consumption can reduce the abundance of Firmicutes and Lactobacillus belonging to Firmicutes, while the abundance of Enterococcus increases significantly. Other studies have also found that, *Verrucomicrobia, Actinobacteria, Corynebacterium, Proteobacteria*, and *Proteobacteria* are rich in the intestines of alcoholic mice.The fecal *Veillonellaceae* and *Megasphera* of patients with alcoholic hepatitis and liver cirrhosis increased significantly. The use of rifaximin can significantly reduce the abundance of gram-negative bacillus *Veillonellaceae*, thereby reducing the incidence of endotoxemia. Yu et al. found that *Turicibacter* is widespread in the intestines of rats with alcoholic liver injury, which is the dominant group. We also found a large amount of *Turicibacter* in the AF group, and the honey group has a lower abundance. The higher abundance of *Prevotellaceae_UCG_001* in MH also appeared in Yi et al.’s study on the influence of acute alcohol intake on the intestinal flora, and the abundance of alcohol intake of *Prevotellaceae_UCG_001* would increase. *Christensenellaceae_R_7_group*, which is also highly abundant in MH, is a beneficial flora.

As functional substances, polyphenols are currently considered to regulate the intestinal flora mainly in two aspects. On the one hand, polyphenols can provide metabolic substrates for intestinal microorganisms and promote the growth and reproduction of probiotics; on the other hand, polyphenols can provide metabolic substrates for intestinal microorganisms; On the one hand, the antibacterial activity of polyphenols can inhibit the growth of harmful bacteria in the intestine and reduce the toxicity caused by pathogenic bacteria. This is mainly because the hydroxyl group of polyphenols can combine with the lipid bilayer of the cell membrane of harmful bacteria to destroy its normal function. It can also destroy the permeability of the cell membrane through the generated hydrogen peroxide, causing the pathogenic bacteria to fail to function normally growth and reproduction; Secondly, polyphenols can form complexes by chelating with metal ions, making the microbial enzyme system unable to catalyze the reaction normally, and even inhibit the enzymatic activity of some enzymes, thereby affecting a series of enzymatic reactions in the intestine.

The present study provides a detailed picture of the phytochemical properties of natural honey from *Triadica Cochinchinensis*, a plant used in traditional Chinese medicine. TCH showed a significant protective effect against alcohol-induced liver disease as well as the activity on maintaining the balance of the microbial ecology. The results suggested that the TCH intervention may benefit the stability of certain gut microbiota, especially in an environment-triggered microbial imbalance, and affect corresponding metabolic pathways, making the contribution to the maintenance of human health.

## Data Availability Statement

The original contributions presented in the study are included in the article/**Supplementary Material**. Further inquiries can be directed to the corresponding author.

## Ethics Statement

The animal study was reviewed and approved by animal ethics review committee of Nanchang University(Permission number: SYXK(G)2015-0002). Written informed consent was obtained from the owners for the participation of their animals in this study.

## Author Contributions

FN and LL conceived this research and designed experiments. JZ and XH participated in the design and interpretation of the data. FN, LL, JZ, SQ, SY, WY, and TL performed experiments and analysis. FN and LL wrote the paper and participated in the revisions of it. All authors contributed to the article and approved the submitted version.

## Funding

This work was supported by the National Natural Science Foundation of China (No. 31772067 and 32001779), Jiangxi Agriculture Research System (No. JXARS-14), China Postdoctoral Science Foundation (No. 2019TQ0137, 2019M662280) and Research Project of State Key Laboratory of Food Science and Technology (SKLF-ZZB-201918).

## Conflict of Interest

The authors declare that the research was conducted in the absence of any commercial or financial relationships that could be construed as a potential conflict of interest.
