# Supplementary material for: Monofloral Triadica Cochinchinensis Honey Polyphenols Improve Alcohol-Induced Liver Disease by Regulating the Gut Microbiota of Mice
Source: Front Immunol. 2021 May 21;12:673903. doi: 10.3389/fimmu.2021.673903 (PMC8175904; doi:10.3389/fimmu.2021.673903)

**Monofloral** **Triadica Cochinchinensis Honey** **Polyphenols Improve** **Alcohol-induced Liver Disease by Regulating the Gut** **Microbiota of Mice**

Liping Luo^ab^, Jinping Zhang^a^, Mingyan Liu^a^, Shengrong Qiu^a^, Shengxiang Yi^a^, Wenjie Yu^a^, Tao Liu^a^, Xueyong Huang^a^, Fangjian Ning^ab*^

^a^ School of Life Sciences, Nanchang University, Nanchang, 330031, China

^b^ State Key Laboratory of Food Science and Technology, Nanchang University, Nanchang, 330031, China

***Corresponding authors:**

Prof. Dr. Fangjian Ning

Email: fangjian-ning@hotmail.com

Tel: +86-0791-83969519; Fax: +86-0791-83969519

Table S 1 Histopathological Assessment Grading

| Hepatic disease | | Scores |
| --- | --- | --- |
| Steatosis | Nonsteatosis | 0 |
|  | Range of steatosis<30% | 1＋ |
|  | Range of steatosis>30% | 2＋ |
|  | Range of steatosis>50% | 3＋ |
|  | Range of steatosis>75% | 4＋ |
| Inflammation | No inflammation present | 0 |
|  | Ballooning degeneration of liver cells<30% | 1＋ |
|  | Ballooning degeneration of liver cells>30% | 2＋ |
|  | Ballooning degeneration of liver cells>50% | 3＋ |
|  | Ballooning degeneration of liver cells>75% | 4＋ |
| Hepatic fibrosis | Non-fibrosis | 0 |
|  | Hepatic fibrosis<30% | 1＋ |
|  | Hepatic fibrosis>30% | 2＋ |
|  | Hepatic fibrosis>50% | 3＋ |
|  | Hepatic fibrosis>75% | 4＋ |

| Parameter | PF | AF |  | PC | LH | MH | HH | FG |
| --- | --- | --- | --- | --- | --- | --- | --- | --- |
| Steatosis | 0 | 4 |  | 1 | 3 | 1 | 2 | 4 |
| Inflammation | 0 | 1 |  | 1 | 0 | 2 | 1 | 2 |
| Hepatic fibrosis | 0 | 0 |  | 0 | 0 | 0 | 0 | 1 |
| Total | 0 | 5 |  | 2 | 3 | 3 | 3 | 7 |

**Figure S1** The BPI of TCH by UPLC-Q-TOF-MS

**
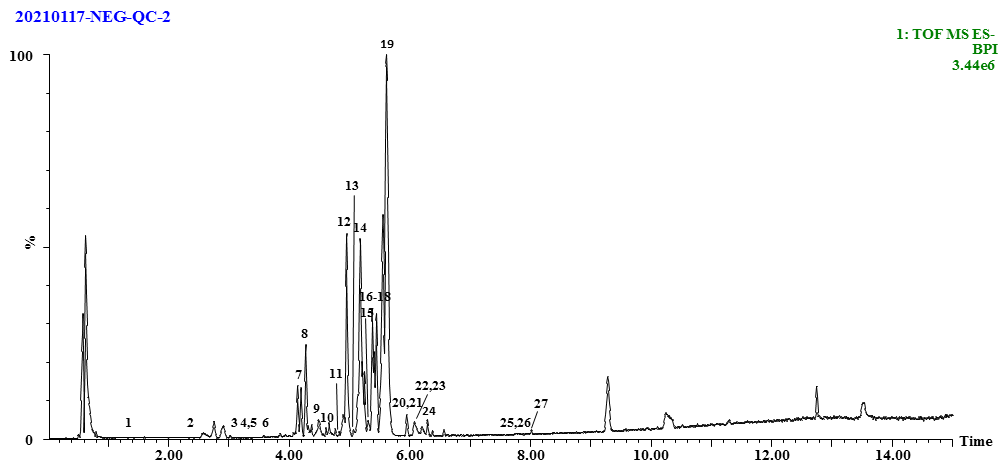
**

**Figure S2.** Comparison of the differences of gut microbiota diversity index among seven groups of mice. Chao index (a), Ace index (b), Shannon index (c) and Simpson index. Data are expressed as mean 土SD. Graph bars with different letters on top correspond to statistically significant results (P<0.05) based on one-way ANOVA analysis, whereas bars with the same letter correspond to results that showed no statistically significant differences ( P>0.05)


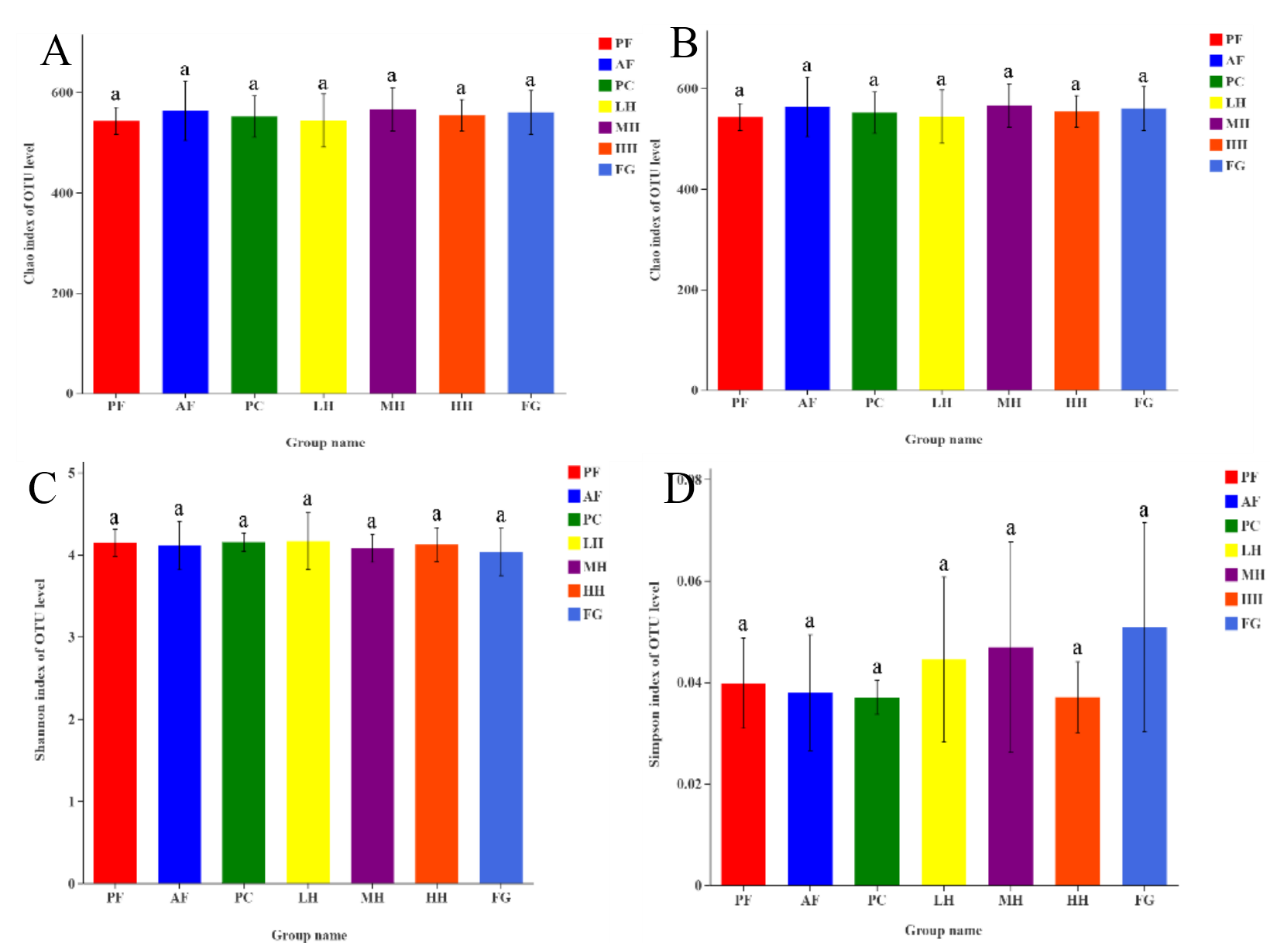


**Figure S3**. LEfSe analyses of gut microbiota in seven groups of mice. Differentially expressed taxa with the LDA scores > 2.0 and adjusted p < 0.05. The taxonomic histograms show the LDA scores calculated for characteristics at the OTU level.


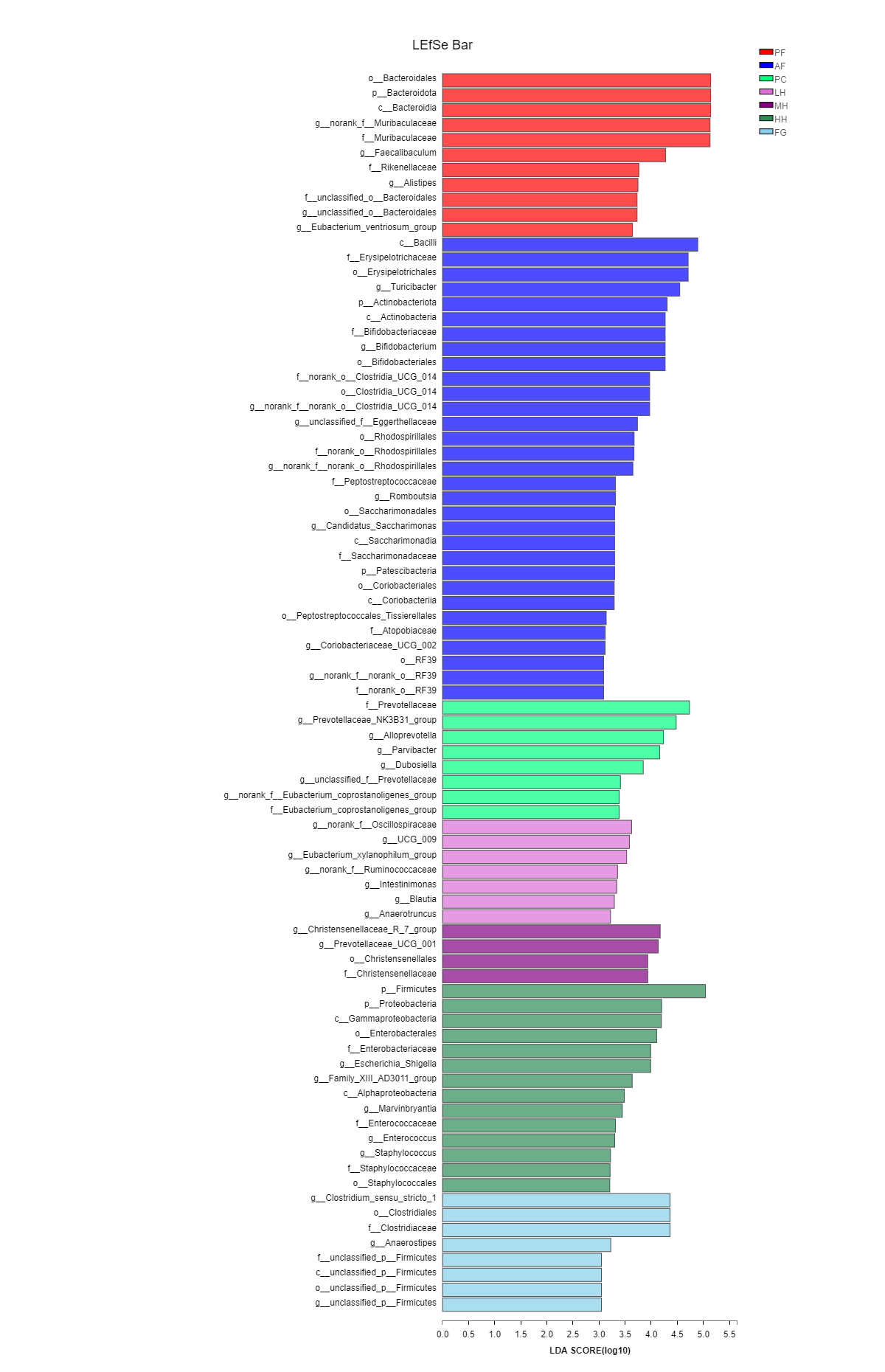

Supplement: Supplementary file 1 [file DataSheet_1.docx]
